# Permanent Draft Genome Sequence for *Frankia* sp. Strain Cc1.17, a Nitrogen-Fixing Actinobacterium Isolated from Root Nodules of *Colletia cruciata*

**DOI:** 10.1128/genomeA.00530-17

**Published:** 2017-06-15

**Authors:** Erik Swanson, Rediet Oshone, Imen Nouioui, Feseha Abebe-Akele, Stephen Simpson, Krystalynne Morris, W. Kelley Thomas, Arnab Sen, Faten Ghodhbane-Gtari, Maher Gtari, Louis S. Tisa

**Affiliations:** a University of New Hampshire, Durham, New Hampshire, USA; b University of North Bengal, Siliguri, India; c Université de Tunis El Manar, Tunis, Tunisia; d Newcastle University, Newcastle upon Tyne, United Kingdom

## Abstract

*Frankia* sp. strain Cc1.17 is a member of the *Frankia* lineage 3, the organisms of which are able to reinfect plants of the Eleagnaceae, Rhamnaceae, and Myricaceae families and the genera* Gynmnostoma* and *Alnus*. Here, we report the 8.4-Mbp draft genome sequence, with a G+C content of 72.14% and 6,721 candidate protein-coding genes.

## GENOME ANNOUNCEMENT

Members of the genus *Frankia* are well known for their ability to form a symbiotic association with a variety of dicotyledonous plants from 8 different families collectively termed actinorhizal plants (1). This interaction results in the formation of a root nodule structure that contains these nitrogen-fixing bacteria. Based on molecular phylogenetic evidence (2–6), *Frankia* consists of 4 major lineages that have been correlated with plant host ranges, and genomes for representatives of each cluster have been sequenced (7). Until recently, *Frankia* strains have not been identified to the species level. Since the sequencing of several *Frankia* genomes, several different species have been and will continue to be recognized (8, 9).

Besides being broad-host-range symbionts, members of *Frankia* lineage 3 exhibit the greatest genetic diversity between strains, have the highest metabolic potential, and possess larger genomes than the other lineages. Many of these strains have adapted to harsh environmental conditions. *Frankia* sp. strain Cc1.17 was isolated from root nodules of *Colletia cruciata* (10). The strain has been investigated for its physiology (11, 12) and is used in genetics studies, including the identification of genetic markers (13, 14) and mutagenesis experiments (15). *Frankia* sp. strain Cc1.17 was sequenced to provide greater insight into this lineage and its interaction with actinorhizal plants. The genome sequence will also be used to assist in the elucidation of lineage 3 diversity, with the goal of identification to the species level.

Sequencing of the draft genome of *Frankia* sp. strain Cc1.17 was performed at the Hubbard Center for Genome Studies (University of New Hampshire, Durham, NH) using Illumina technology techniques (16). A standard Illumina shotgun library was constructed and sequenced using the Illumina HiSeq 2500 platform, which generated 15,413,374 reads (260-bp insert size) totaling 3,945 Mbp. The Illumina sequence data were trimmed by Trimmonatic version 0.32 (17) and assembled using SPAdes version 3.5 (17) and ALLPaths-LG version r52488 (18). The final draft assembly for *Frankia* sp. strain Cc1.17 consisted of 195 contigs, with an *N*_50_ contig size of 118.5 kb and 356.3× coverage of the genome. The final assembled genome contained a total sequence length of 8,361,025 bp, with a G+C content of 72.14%.

The assembled *Frankia* sp. strain Cc1.17 genome was annotated via the NCBI Prokaryotic Genome Annotation Pipeline (PGAP) and resulted in 6,721 candidate protein-coding genes. Bioinformatic analysis of this genome using the antiSMASH program (19, 20) revealed the presence of high numbers of secondary metabolic biosynthetic gene clusters, including 4 nonribosomal peptide synthetase, 12 polyketide synthase, 4 terpene, 1 bacteriocin, 3 lantipeptide, and 1 siderophore cluster. This large number is consistent with previous results with other *Frankia* lineage 3 strains (7).

### Accession number(s).

This whole-genome shotgun sequence has been deposited at DDBJ/EMBL/GenBank under the accession number MBLM00000000. The version described in this paper is the first version, MBLM01000000.
